# Merging Two Models of One-Dimensional Convolutional Neural Networks to Improve the Differential Diagnosis between Acute Asthma and Bronchitis in Preschool Children

**DOI:** 10.3390/diagnostics14060599

**Published:** 2024-03-12

**Authors:** Waleed Salih, Hakan Koyuncu

**Affiliations:** 1 Information Technologies Department, Altinbas University, Istanbul 34217, Turkey; waleed.salih@ogr.altinbas.edu.tr; 2 Computer Engineering Department, Altinbas University, Istanbul 34217, Turkey

**Keywords:** acute asthma and bronchitis, differential diagnosis, one-dimensional convolutional neural network

## Abstract

(1) Background: Acute asthma and bronchitis are common infectious diseases in children that affect lower respiratory tract infections (LRTIs), especially in preschool children (below six years). These diseases can be caused by viral or bacterial infections and are considered one of the main reasons for the increase in the number of deaths among children due to the rapid spread of infection, especially in low- and middle-income countries (LMICs). People sometimes confuse acute bronchitis and asthma because there are many overlapping symptoms, such as coughing, runny nose, chills, wheezing, and shortness of breath; therefore, many junior doctors face difficulty differentiating between cases of children in the emergency departments. This study aims to find a solution to improve the differential diagnosis between acute asthma and bronchitis, reducing time, effort, and money. The dataset was generated with 512 prospective cases in Iraq by a consultant pediatrician at Fallujah Teaching Hospital for Women and Children; each case contains 12 clinical features. The data collection period for this study lasted four months, from March 2022 to June 2022. (2) Methods: A novel method is proposed for merging two one-dimensional convolutional neural networks (2-1D-CNNs) and comparing the results with merging one-dimensional neural networks with long short-term memory (1D-CNNs + LSTM). (3) Results: The merged results (2-1D-CNNs) show an accuracy of 99.72% with AUC 1.0, then we merged 1D-CNNs with LSTM models to obtain the accuracy of 99.44% with AUC 99.96%. (4) Conclusions: The merging of 2-1D-CNNs is better because the hyperparameters of both models will be combined; therefore, high accuracy results will be obtained. The 1D-CNNs is the best artificial neural network technique for textual data, especially in healthcare; this study will help enhance junior and practitioner doctors’ capabilities by the rapid detection and differentiation between acute bronchitis and asthma without referring to the consultant pediatrician in the hospitals.

## 1. Introduction

Acute bronchitis and asthma are common diseases that affect lower respiratory tract infections (LRTI) in children worldwide and cause coughing, inflammation, and airway irritation; these diseases can occur at any age and occur mostly in childhood due to their rapid spread [1]. Most people confuse acute asthma and bronchitis because they have overlapping symptoms (runny nose, shortness of breath, wheezing, and cough). Therefore, practitioners and junior doctors face difficulty differentiating between them, especially in urgent cases within emergency departments [2]. Acute asthma occurs in childhood by viral infection or some allergens as the result of air irritants, such as fumes and dust mites, or smoke and strong odors or perfumes; sometimes the causes may be deterioration in the case of children through influenza, sinusitis, or upper respiratory infection, as well some cases due to family history of the children [3]. Symptoms of acute asthma start with shortness of breath, wheezing, cough, and eczema, and symptoms differ according to age and immunity of the child; it occurs as a result of the contraction of muscles where breathing becomes difficult because the mucus production surrounding the airways increases, causing an obstruction. Children’s illnesses can differ in terms of their severity and length; in some cases, acute asthma episodes last only a few minutes if they are moderate and extend for several hours or days if they are severe; therefore, it is necessary to visit hospital due to the life-threatening risk to children, where pediatricians can be identified of acute asthma based on examination and medical history of the child [4]. Acute bronchitis is a viral infection (self-limited) of the upper airways, where a cough appears in infection cases, which is the first of the diagnostic symptoms of the disease and can be diagnosed after excluding other respiratory diseases, pain bronchiolitis, and colitis: it is considered the most common clinical disease in the United States, Africa, and Asia [5]. Acute bronchitis can be caused by Viruses or uncommon bacterial infections, and sometimes bacteria and irritant allergens such as polluted air, smoke, and dust are reasons for acute bronchitis; it is estimated that (5%) of the general population is infected with acute bronchitis each year, commonly occurring during the flu season in winter and autumn. Acute bronchitis can occur as a result of upper respiratory tract infections; symptoms of the disease include productive cough, shortness of breath, wheezing, low-grade fever, headache, runny nose, and others. Usually, the cough continues after acute bronchitis for 10–20 days and it sometimes lasts for four weeks, with the average cough lasting for 18 days after acute bronchitis. Acute asthma is diagnosed as acute bronchitis for one-third of patients who have an acute cough misdiagnosed [6]. Acute asthma and bronchitis have various causes, but their symptoms overlap, so when such symptoms occur, it is necessary to visit the emergency department to receive proper treatment for the case that the pediatrician diagnoses; if cases last without appropriate treatment, they may lead to chronic disease infection. A manual diagnosis to differentiate between acute bronchitis and acute asthma is cumbersome and takes a long time due to overlapping symptoms between the two diseases, reaching 12 clinical features, to obtain an accurate clinical examination [7,8]. Large amounts of medical data can be dealt with through a deep learning approach to speed up processing, find the patterns between different and multiple sources, and increase predictive power; they can also reveal the complexity and differentiate between nonlinear sources of latent variance related to illness and early disease detection, especially diseases with overlapping symptoms [9]. Moreover, deep learning models can be trained in novel ideas and mechanisms, through which new patterns and features of data can be discovered, in addition to motivating healthcare specialists to make appropriate decisions in early diagnosis and treatment of infected children without referring to consultants and improving access to healthcare services provided. The proposed study aims to develop deep learning algorithms and discover novel features by merging two one-dimensional convolutional neural network models to improve differential diagnosis between acute asthma and bronchitis, evaluate the proposed model’s performance, and compare the results with other models.

## 2. Literature Review

Katy Stokes et al. (2021) proposed machine learning methods to diagnose pneumonia and bronchitis for a group of middle-income patients in Sarajevo Hospital and through which data were collected for the infected patients, which consisted of 4500 real cases that specialist doctors clinically examined. The dataset, which consisted of 3000 cases of pneumonia and 1500 cases of bronchitis, ran from October 2017 to December 2018; it was divided into 60% for training and 40% for testing, and during the study, three algorithms for ML were tested (logistic regression, decision tree, and support vector machine (SVM). After being developed by researchers and comparing them, the decision tree achieved the highest accuracy of 93% with AUC [2]. Yao Tong et al. (2022) performed a study to predict the future continuing of care (COC) by developing machine learning models for asthmatic patients and discovering associated factors; the dataset consisted of 31,724 cases of patients who received healthcare from Washington University of Medicine for nine years, from January 2011 to December 2018, where the construction of the machine learning model relied on examining 128 features with 10-fold cross-validations of the model. Several models of ML have been used: baseline KNN, naive Bayes, (SVM), random forest, and XGBoost (extreme gradient boosting), where the highest accuracy was 88.20% for the XGBoost model; however, they used the ROC, which was 0.96%, and the average F1-score of 0.86%, and the model can facilitate future clinical decisions and improve hospital management outcomes [10]. Yoshihiko Raita et al. (2020) proposed a study aimed at developing ML models to predict the severity of bronchiolitis in infants under one year, where real data of 1016 prospective cases were collected over three years (2011 to 2014) and distributed across three seasons (November to April). The cases were clinically examined by specialized doctors following the guidelines of the American Academy of Pediatrics; the most common symptom was shortness of breath (SOB), the average age patients was 3.2 months, with females accounting for 42%, and the length of stay in the hospital for the disease cases was 0 to 60 days. Researchers developed four machine learning models: Lasso regularization with logistic regression, elastic net regularization with logistic regression, random forest, and gradient boosted decision tree. After comparing the models, gradient boosted decision tree outperformed with an accuracy of 95% and an AUC of 88% [11]. In 2015, Kiranyaz et al. presented the first 1D-CNN and applied it to the ECG signals of affected patients, as it was small and could adapt to the data type. In addition, the real time application was low in cost; unlike the 2D-CNN, which contains operational time complexity and large bifurcation, 1D-CNN can be easily trained to achieve high performance by providing the minimum number of complex computational operations and solving classification problems such as heart diseases and respiratory diseases in children. It can also merge the features of extension and application and make it in one adaptive environment [12]. LSTM is an advanced version of the recurrent neural network (RNN) standard, which suffers from the vanishing gradient problem because it contains short-term memory, especially with long sequential data; therefore, LSTM can insert large blocks of memory and move information forward and keep it from previous sequential parts instead of connected hidden units, thus solving the vanishing gradient problem [13]. In 2020, Chen et al. proposed a method that merges two models of 1D-CNN with LSTM for arrhythmia classification by automatically identifying six ECG signals; researchers used a dataset from the PhysioNet website. In addition, three sets of ECG data were used for comparison and evaluation. The final results showed an accuracy of 99.32% [14]. In 2021, Muhammad Al-Khatib and others presented a method based on merging two convolutional neural networks, 1D-CNN and 2D-CNN, to detect nerves in ultrasound images, where detecting nerves is one of the most difficult tasks faced by anesthesiologists. The final results of the merged model showed high accuracy in the experiments and outperformed other traditional CNNs by 10% [15]. Mohamed G. El-Shafiey and his colleagues (2021) proposed to build a hybrid deep learning model by using bidirectional LSTM with 1D-CNN to predict heart disease through binary classification for the presence or absence of illness. The researchers used the dataset from the UCI ML library, which contains two parts: the first section of Statlog contains 120 cardiac records, while Cleveland contains 150 records. The dataset is divided into 70% training and 30% testing. The final results of the proposed approach achieved in binary classification were an accuracy of 89.01% and 82.72%, respectively, for the Cleveland and Statlog dataset [16].

Despite the size of the actual dataset being good, it was not close to avoiding oversampling. In addition, the researchers did not use other models to find a higher accuracy [2]. The accuracy was acceptable, and the researchers should have trained other types of deep learning algorithms and discovered other features [10]. The idea for researchers was good because they developed models and obtained high accuracy after collecting real prospective cases, but the percentage of AUC does not correspond to some degree of accuracy [11]. The researchers used deep learning algorithms and merging techniques; the accuracy of the results was high [12,13,14,15].

In this study, some gaps will be highlighted and processed; close samples were taken from both diseases to avoid sampling. The number of cases of acute asthma was 248, and of bronchitis, 264. The affected cases were examined exclusively by a consultant pediatrician to avoid misdiagnosis. We compare the accuracy of the proposed model’s performance with other modern models.

## 3. Dataset 

### 3.1. Dataset Description 

A consultant pediatrician collected the real dataset in Iraq at Fallujah Hospital for Women and Children from 1 March 2022 to June 2022, where 512 prospective cases (248 acute asthma and 264 acute bronchitis) were examined. Each case contained 12 clinical features identified by the pediatrician, as in Table 1. 

### 3.2. Influencing Factors 

The factors influencing the number of cases are weather fluctuations that the city experienced during the period, such as high and low temperatures, air pollution, and dust. Acute bronchitis increased in March and April when temperatures were low, while acute asthma increased in May and June when the city weather was dusty [17,18]. Table 2 shows the number of cases distributed by month.

### 3.3. Dataset Analysis

**Figure 1:**
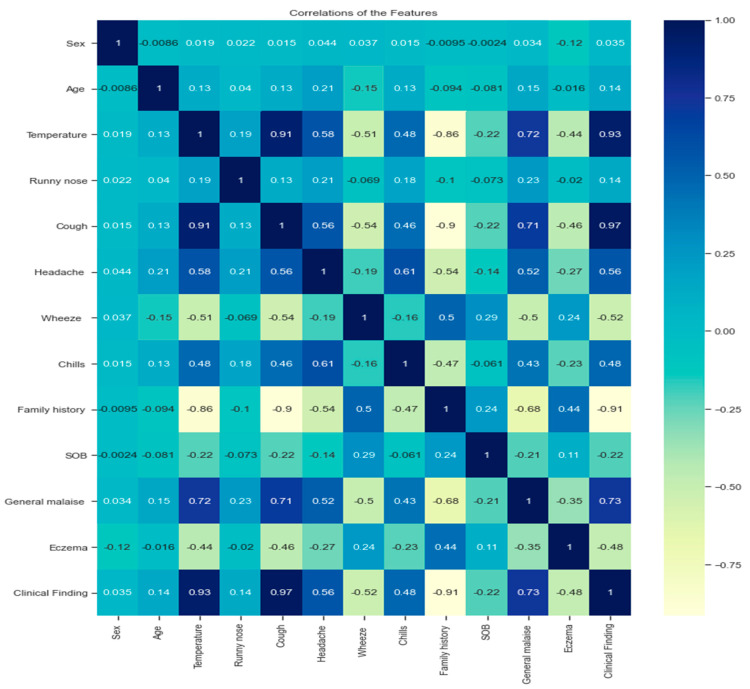
Correlation matrix of the dataset.

Data analysis is necessary to know the relationship between positive or negative variables and the type of relationship: linear or nonlinear. This study used the correlation matrix in Figure 1 to determine the most closely related pairs between variables through heatmaps of correlation to find the strength of the relationship between the numerical variables of the dataset as follows:

- A strong positive correlation between the variables (cough, temperature; cough, general malaise; temperature and general malaise) can be explained by the existence of multiple linear relationships between the variables because the Pearson correlation coefficient is more significant than 0.7.
- A strong negative correlation between variables (temperature, family history; cough, family history).
- There is a strong positive correlation between some variables and the target variables (clinical findings), such as temperature, cough, and general malaise. Their effect on the target was more significant than 0.7.
- A strong negative correlation exists between family history and clinical finding.

Analyzing the dataset using the correlation matrix is very important for physicians. They can find the relationship between two features and make appropriate medical decisions. It also helps to reduce the dimensions of extensive medical data by dropping one of the features with a strong positive relationship to prevent repetition that may affect overfitting and thus affect the performance of the final model [19,20].

### 3.4. Preprocessing Dataset

Data preprocessing included cleaning the data and verification with the consultant pediatrician, removing redundant values, selecting appropriate data that affect the classification process, as 12 standard clinical features between acute bronchitis and asthma were maintained; also, converting the text data into numeric data, then standardizing it within the range (−1, 1) to be easy. Finally, the dataset was divided into 70% training data and 30% testing; the cross-validation technique implemented k-10-fold cross-validation.

## 4. Methods

### 4.1. Proposed Method

This study presents two identical models for a one-dimensional convolutional neural network and then merges both models to obtain a merged model of 2-1D-CNNs, as shown in Table 3. Also, we merge 1D-CNNs with long short-term memory to obtain a hybrid model (1D-CNNs + LSTM), as in Table 4.

### 4.2. Flowchart Architectures

**Figure 2:**
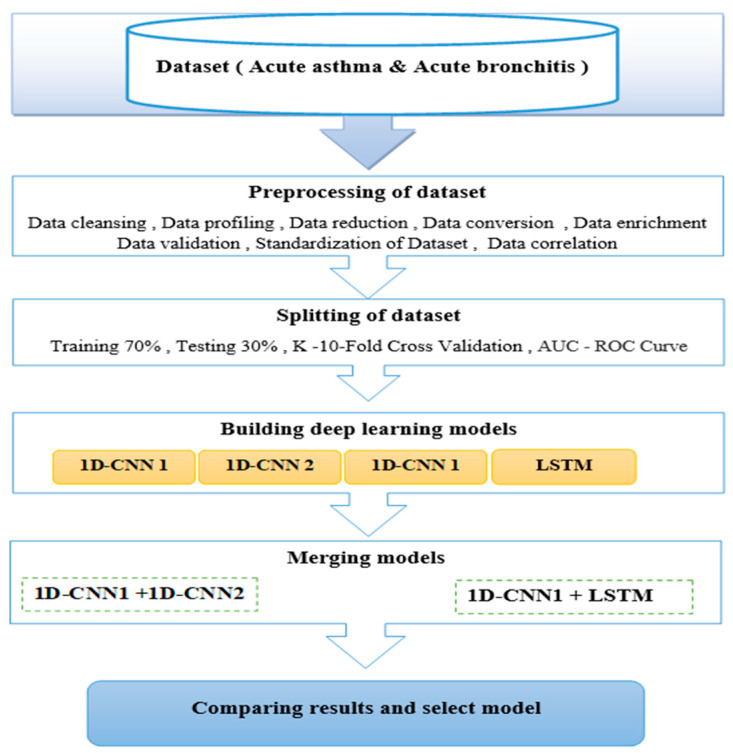
Flowchart architecture.

Figure 2 shows the flowchart architecture and techniques used for the proposed method.

### 4.3. One-Dimensional Neural Networks (1D-CNNs)

Convolutional neural networks are designed to work exclusively with two-dimensional data such as images, video, and X-rays, so they are referred to as 2D-CNNs. In 2015, the first one-dimensional neural network was operated by Kiranyaz et al. It was applied directly to patients’ ECG signals. It was compacted and adaptable in a short time. There is a significant difference between 1D and 2D convolutions in computational complexities, i.e., the image with N × N dimensions, which convolves with K × K kernel, will have computational complexity of ~O($N^{2}K^{2}),$ while in the 1D convolution (with the same N and K dimensions), it will have a computational complexity of ~O(NK); thus, the computational complexity of 1D-CNNs is much lower than the 2D-CNN with the same configuration, hyperparameters, and network. The 1D-CNNs can be trained relatively quickly with any CPU application on a standard computer, unlike deep 2D-CNNs which require particular hardware setups such as cloud computing or GPU farms. Due to 1D-CNNs’ low computational requirements, it well-suited for low-cost and real-time applications, especially on hand-held devices or mobile, therefore 1D-CNNs have made significant progress in healthcare, especially in early diagnosis of diseases, additionally to other applications of energy [12]. Each 1D-CNN can be expressed as the 1D forward propagation (1-FP) in Equations (1) and (2) [21].

(1) $$x_{k}^{l}=b_{k}^{l}+\sum_{i=1}^{Nl-1}ConvID\left(W_{ik}^{l-1},S_{i}^{l-1}\right)$$

where $x_{k}^{l}$ refers to the input, $b_{k}^{l}$ represents a bias of the Kth neuron at layer l, $S_{i}^{l-1}$ is the output of the ith neuron at layer l − 1, $W_{ik}^{l-1}$ is a kernel from the ith neuron at layer l − 1 to the Kth neuron at layer l, the dimension of the input array $x_{k}^{l}$ is less than the dimension of the output arrays. Conv1D is used to perform invalid(1D) convolution without zero-padding, and can be expressed as the intermediate output, $y_{k}^{l}$, by passing the input $x_{k}^{l}$ through the activation function f (.) as given in Equation (2)

(2) $$y_{k}^{l}=f\left(x_{k}^{l}\right)\mathrm{and}S_{k}^{l}=y_{k}^{l}↓SS$$

In Equation (2), SS refers to a down sampling operation with a scalar factor, SS, and $S_{k}^{l}$ stands for the output of the Kth neuron to the layer l.

### 4.4. Long Short-Term Memory (LSTM)

**Figure 3:**
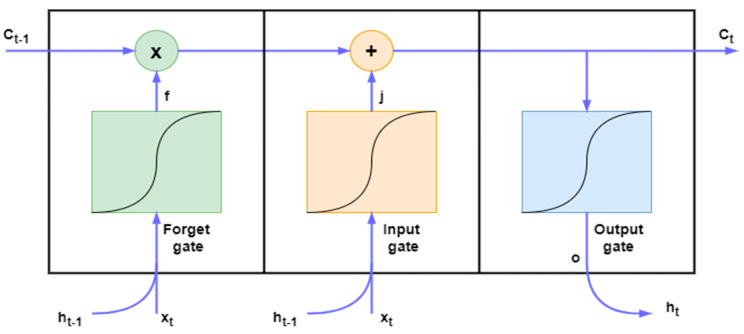
LSTM memory cell.

It is an artificial neural network and an advanced version of RNN that suffers from short-term memory because of the vanishing gradient problem; LSTM refers to the RNN and has both long-term memory and short-term memory, the connection weights and biases change in the network once per episode of training; similar to how physiological changes in synaptic strengths store long-term memory, the activation patterns change in the network once per time step, analogous to the moment-to-moment change in electric firing patterns in the brain store short-term memories, the architecture of LSTM aims to supply a short-term memory for RNN which can last thousands of time steps, hence the name long short-term memory [13]. LSTM consists of three doors: input gate, output gate, and forget gate. It is a sigmoid activation function as in Figure 3, in Equations (3)–(8) where (W) represents weight matrices, (b) represents the input bias vector, (C_t_) is a cell state, (i) is the input gate, (f) means the forget gate, (o_t_) is the output gate, and extracellular (tanh) is the activation function. The output layer is the final layer that is used to estimate sensitivity. Equation (6) represents the input essential of LSTM architecture, Equation (7) the forget gates, Equation (8) the output, and memory cells is Equation (3) [22].

(3) $$f_{t}=\sigma \left(W_{f}\cdot \left[h_{t}-1’x_{t}\right]+b_{t}\right)$$

(4) $$i_{t}=\sigma \left(W_{i}\cdot \left[h_{t}-1’x_{t}\right]+b_{i}\right)$$

(5) $${\tilde{c}}_{t}=\tanh(W_{c}\cdot \left[h_{t}-1’x_{t}\right]+b_{c})$$

(6) $$C_{t}=f_{t}\times c_{t-1}+i_{t}\times {\tilde{c}}_{t}$$

(7) $$o_{t}=\sigma \left(W_{o}\cdot \left[h_{t}-1’x_{t}\right]+b_{o}\right)$$

(8) $$h_{t}=o_{t}\times \tanh\left(C_{t}\right)$$

### 4.5. Merge 2-1D-CNNs

To ensure the merging technique of two models of (1D-CNNs), the input data for both models were represented as a one-dimensional array (12, 1); both models were built by three one-dimensional convolutional layers (32, 64, 128) respectively with a kernel size of 2 in each convolution layer to improve output in the appropriate way through extracting features from the 1-D array which was multiplied by the input, due to the filter moving only in one direction. Thus, the output is 1D, then drops out after layers at different rates (0.1, 0.2, 0.5) according to the size of the convolutional layer to avoid overfitting [23]. The Rectified linear Function (Relu) was used with each convolutional layer because it is easy to calculate and only compares the input with the zero value, as in Equation (9).

(9) $$R\left(x_{i}\right)=\left\{\begin{array}{c} 0\;\;\left(x_{i}\leq 0\right)\;\\ x_{i}\;\;\left(x_{i}>0\right) \end{array}\right.$$

The max pooling layer was used with both models of two sizes; in the first model, it was after three convolution layers, while in the second model, it was after each convolution layer to extract the features, thus reducing contrast, size of data, and the number of parameters through taking the largest value and keeping it in a pooling rectangle, we added a fully connected layer (flatten) to both models where the input layer turns the outputs of the previous layers to a single vector, in addition, we added a first fully connected layer with dense (128) and dropout (0.3) in the first model and (0.5) in the second model to apply the weights to predict it, and we added a fully connected output layer (Dense 1) at the end of the first model with the sigmoid activation function to give us results of zero or one due to the problem in this paper being a binary classification, the sigmoid function is expressed in Equation (10) [24].

(10) $$s\left(x\right)=1/(1+e^{-x})$$

**Figure 4:**
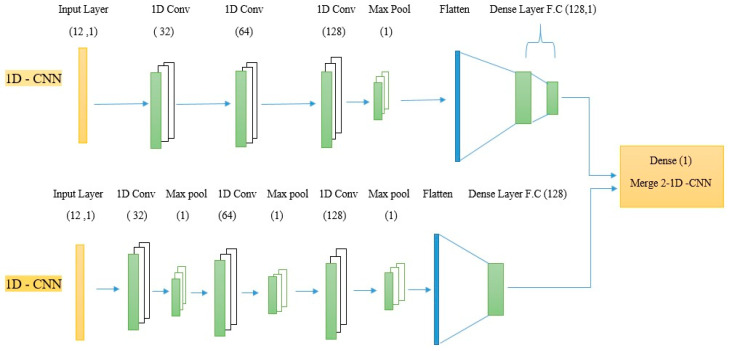
Merge 2-1D-CNNs.

Finally, we merged the first and second models in 1D-CNNs by adding an output layer (Dense 1) with a sigmoid activation function, as in Figure 4.

### 4.6. Merge 1D-CNNs + LSTM

**Figure 5:**
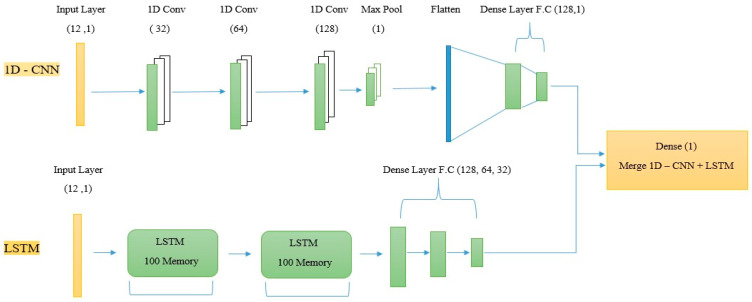
Merge 1D-CNNs + LSTM.

This method merges the first model of a one-dimensional convolutional neural network with long-term memory. The LSTM model created 100 memory units, added them to return sequences, and returned the hidden state as the output for each input time step; the second layer also contained 100 memory units with Relu. After each layer, a dropout of 0.2 was added to reduce overfitting. We distributed three fully connected layers with LSTM layers (128, 64, 32) with a dropout of (0.25, 0.2, 0.1), respectively, to avoid overfitting; also, the sigmoid activation function was used to obtain a classification of either zero or one. After constructing the two models, we merged them into one hybrid model (1D-CNN + LSTM) by adding an output layer (Dense 1) with a sigmoid activation function, as in Figure 5.

## 5. Results and Discussion

The models were trained on the Windows 10 operating system. Python (3.8.8) was the programming language used with Jupyter Notebook, with Visual Studio Code (VSC) TensorFlow used as a deep learning framework. In this study, the parameters were trained for both methods. Cross-entropy loss was selected because the problem is a binary classification, where the optimizer is Adam. AUC (area under the curve) represents the measure or degree of separability. It was applied to the dataset to tell us about the model’s ability to distinguish between categories. The higher the AUC is for predicting 0 and 1, the better the model’s performance at differentiating between acute asthmas. Stratified cross-validation ensured equal and identical distribution of subgroups during training, thus reducing oversampling and improving sample accuracy representation. The models were tested on the test data through 50 epochs, and the model’s test accuracy on the training and testing dataset and the loss function was noted. Early stopping was used to prevent overfitting.

### 5.1. Results of Merging 2-1D-CNNs

**Figure 6:**
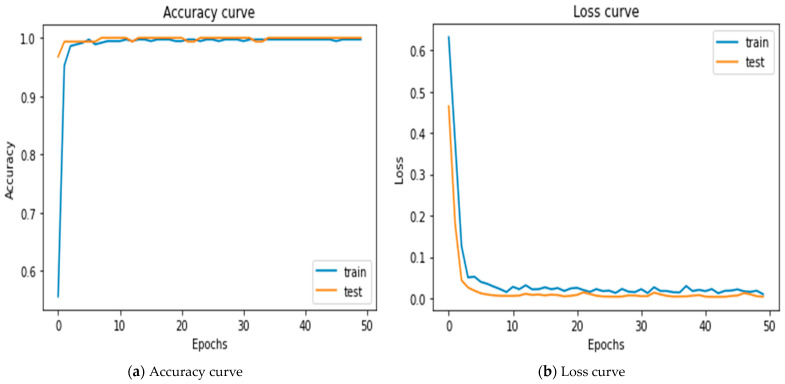
Accuracy curve and loss for merging 2-1D-CNNs.

In order to test the accuracy to differentiate between acute asthma and bronchitis by using different methods, we merged 2-1D-CNN models to obtain a rate accuracy of 99.72 with AUC of 1.0, and test loss for the model of 0.0039. Figure 6a shows that the training accuracy increases continuously to reach strong convergence with the test accuracy, which also began to increase by a high rate, thus avoiding overfitting in the model. Figure 6b shows that the training loss began to decrease at a rate higher than the test loss to reach its final value.

### 5.2. Results of Merging 1D-CNNs + LSTM

**Figure 7:**
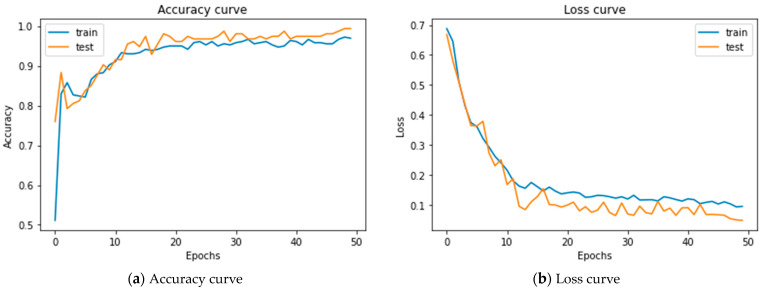
Accuracy curve and loss for merging 1D-CNNs + LSTM.

This method used the same parameters in merging two one-dimensional neural networks. The accuracy rate was 99.35, with an AUC of 99.96, and test loss for the model was 0.048. Figure 7a shows that the training and test accuracy increased, decreased a little, and converged with the test accuracy to reach the final value. Figure 7b shows that the training loss and test loss began to decrease with the same trends to reach their final value.

### 5.3. Results Comparison

This section compares the methods used to differentiate between acute asthma and bronchitis regarding area under the curve, number of parameters, test loss, and accuracy for models, as shown in Table 5.

**Figure 8:**
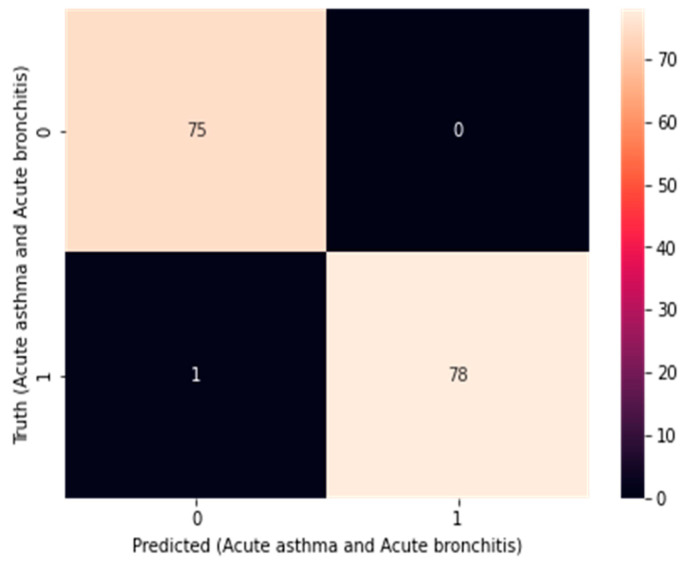
Confusion matrix for merging 2-1D-CNNs.

**Figure 9:**
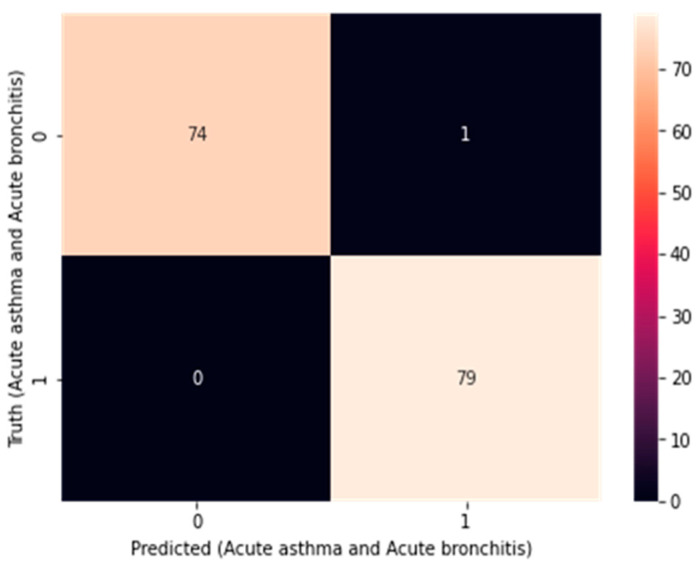
Confusion matrix for merging 1D-CNNs + LSTM.

After testing and verifying the accuracy of these models, the confusion matrix was calculated to merge 2-1D-CNNs, as in Figure 8, and merge 1D-CNN + LSTM, as in Figure 9, and the specificity and sensitivity were applied for the dataset, which mathematically represented the accuracy of a test which refers to the presence or absence case, as in Equations (11)–(13).

(11) $$Accuracy=\frac{TN+TP}{TN+FP+FN+TP}$$

(12) $$Sensitivity=\frac{TP}{TP+FN}$$

(13) $$Specificity=\frac{TN}{TN+FP}$$

Here, sensitivity indicates the possibility of a positive test (actual positive rate) if it is genuinely positive, while specificity indicates the possibility of a negative test (actual negative rate) if it is genuinely negative. We applied the precision (also called the value of positive predictive) as part of the relevant cases among the retrieved cases. At the same time, recall (also referred to as sensitivity) is the part of retrieved relevant cases. Therefore, recall and precision are based on relevance, as in Equations (14) and (15). Thus, there is a need to use F1-score for evaluating performance through metrics required to predict performance, as in Equation (16) [25,26]. Table 6 shows the results of the model performance.

(14) $$Precision=\frac{TP}{TP+FP}$$

(15) $$Recall=\frac{TP}{TP+FN}$$

(16) $$F1\text{-}score=2\times \frac{Precision\times Recall}{Precision+Recall}$$

### 5.4. Comparison of Existing Results with Previous Studies’ Results

This section compares current and previous studies, as in Table 7.

## 6. Conclusions

In this study, a novel method was presented to improve differential diagnosis between acute asthma and bronchitis in preschool children by merging 2-1D-CNNs for a dataset consisting of 512 prospective cases collected by the consultant pediatrician at Fallujah Teaching Hospital for women and children during the period from March 2022 to June 2022. The preprocessing of the dataset included cleaning, removing redundant values, analysis, and verification with a pediatrician to maintain 12 clinical features for each case. The dataset was split into training data of 70% and test data of 30%. The cross-validation technique implemented 10 k-fold to estimate model performance during training, evaluated it iteratively, and reduced bias compared with split data training and testing. The first model merged 2-1D-CNNs. Due to 1D-CNNs’ low computational requirements, it is well suited for low-cost and real-time applications, especially on handheld devices or mobile; in addition to that, it can be easily trained, unlike 2D-CNNs, which require a particular hardware setup. The second model merged 1D-CNNs + LSTM. The same parameters were used in both models: rectified linear function (Relu), because it is easy to calculate and involves comparing only the input with the value zero; and sigmoid function, because the problem is a binary classification. AUC (area under the curve) represents the measure or degree of separability. It was applied to the dataset to tell us about the model’s ability to distinguish between categories; the percentage in the first model was 1.0 and the second was 99.96%. Cross-entropy loss was selected, and the optimizer was Adam. Both models were tested through 50 epochs. Note that the models tested accuracy on the training and testing with the loss function and used early stopping to prevent overfitting. The final results show that the merge of 2-1D-CNNs had an accuracy of 99.72 with a test loss of 0.0039, compared to the merge of 1D-CNNs + LSTM, which had an accuracy of 99.44 with a test loss of 0.048. Thus, the first model can be adopted as a binary classifier to improve differential diagnosis in this study. Finally, this study can be converted into an intelligent application that helps junior and practitioner doctors quickly detect diseases and differentiate between them, especially in hospital emergency halls.

## Figures and Tables

**Table 1:** Clinical features.

Features	Acute Asthma	Acute Bronchitis
Sex	Male/Female	Ma/leFemale
Age	Under six years	Under six years
Temperature	Normal	Low-grade fever
Runny nose	+ve/−ve	+ve
Headage	−ve	+ve
Cough	+ve/dry	+ve/productive
Wheeze	+ve	+ve/−ve
Chills	−ve	+ve/−ve
Family history	+ve	−ve
Shortness of breath	+ve	+ve
General malaise	−ve	+ve
Eczema (allergic)	+ve	−ve

Notes: (+ve) represents presence and (−ve) is absence; there is overlap in the symptoms for both acute bronchitis and acute asthma such as cough, wheeze, runny nose, and shortness of breath; some cases were not stable during the clinical examination and were variable according to immunity and environment of the child.

**Table 2:** Number of cases.

Month	Acute Asthma	Acute Bronchitis
March	39	94
April	63	78
May	77	49
June	69	43
Sum	248	264
Percentage Values	48.4375%	51.5625%

**Table 3:** Proposed model of 2-1D-CNNs.

Layers	Number Neurons	Kernel Size	Activation Function
1D Conv	32	2	Relu
1D Conv	64	2	Relu
1D Conv	128	2	Relu
1D pool	1	-	-
Flatten	-	-	-
Dense	128		Relu
Dense	1	-	Sigmoid
1D Conv	32	2	Relu
1D pool	1	-	-
1D Conv	64	2	Relu
1D pool	1	-	-
1D Conv	128	2	Relu
Flatten	-	-	-
Dense	128	-	Relu
Dense	1	-	Sigmoid

**Table 4:** Proposed model of 1D-CNNs + LSTM.

Layers	Number Neurons	Kernel Size	Activation Function
1D Conv	32	2	Relu
1D Conv	64	2	Relu
1D Conv	128	2	Relu
1D pool	1	-	-
Flatten	-	-	-
Dense	128		Relu
Dense	1	-	Sigmoid
LSTM	100		-
LSTM	100	-	Relu
Dense	128	-	Relu
Dense	64	-	Relu
Dense	32	-	Relu
Dense	1	-	Sigmoid

**Table 5:** Results comparison.

Classifier	AUC	Parameters	Test Loss	Accuracy
1D-CNN	99.32	80.449	0.043	99.35
LSTM	99.86	144.497	0.056	98.701
1D-CNN + LSTM	99.96	312.979	0.048	99.44
1D-CNN + 1D-CNN	1.0	336.963	0.0039	99.72

**Table 6:** Model performance.

Classifier	Sensitivity	Specificity	Precision	F1-Score
1D-CNNs + LSTM	1.0	98.73	99	99
2-1D-CNNs	98.66	1.0	99	99

**Table 7:** Comparison with previous results.

References	Classifier	Accuracy %
[2]	Decision Tree	93
[10]	XGBoost	88.20
[11]	GBDT	95
[15]	1D-CNN + LSTM	99.32
Proposed Method	2-1D-CNNs	99.72

## Data Availability

Dataset available on request from the authors.
